# Development of a Positive Selection High Throughput Genetic Screen in *Dictyostelium discoideum*

**DOI:** 10.3389/fcell.2021.725678

**Published:** 2021-08-13

**Authors:** Felicia N. Williams, Yumei Wu, K. Matthew Scaglione

**Affiliations:** ^1^Department of Molecular Genetics and Microbiology, Duke University, Durham, NC, United States; ^2^Department of Neurology, Duke University, Durham, NC, United States; ^3^Duke Center for Neurodegeneration and Neurotherapeutics, Duke University, Durham, NC, United States

**Keywords:** *Dictyostelium discoideum*, 5-FOA, polyglutamine, protein aggregation, genetic screen

## Abstract

The cellular slime mold *Dictyostelium discoideum* is a powerful model organism that can be utilized to investigate human health and disease. One particular strength of *Dictyostelium* is that it can be utilized for high throughput genetic screens. For many phenotypes, one limitation of utilizing *Dictyostelium* is that screening can be an arduous and time-consuming process, limiting the genomic depth one can cover. Previously, we utilized a restriction enzyme-mediated integration screen to identify suppressors of polyglutamine aggregation in *Dictyostelium*. However, due to the time required to perform the screen, we only obtained ∼4% genome coverage. Here we have developed an efficient screening pipeline that couples chemical mutagenesis with the 5-fluoroorotic acid counterselection system to enrich for mutations in genes of interest. Here we describe this new screening methodology and highlight how it can be utilized for other biological systems.

## Introduction

Model organisms like *Dictyostelium discoideum* (*Dictyostelium*) offer numerous advantages for identifying key components of cellular systems. *Dictyostelium* is particularly useful for these types of studies because a number of different genetic screening technologies have been developed for *Dictyostelium* biology (Bozzaro, 2019). For example, restriction enzyme-mediated integration (REMI) enables screens that rely on disrupting gene function *via* integration of plasmid into the *Dictyostelium* genome (Guerin and Larochelle, 2002). Affected genes can be identified by extracting the plasmid from the genome with restriction enzymes and sequencing retained pieces of genomic DNA to identify disrupted genes (Guerin and Larochelle, 2002). Alternatively, mutagenesis using a chemical mutagen such as *N*-methyl-*N*′-nitro-*N*-nitroguanidine (NTG) can also introduce mutations into the *Dictyostelium* genome that can later be identified by whole-genome sequencing (Li et al., 2016). With these assays, one can rapidly generate mutations at near genome saturation. However, screening randomly mutated cells to identify phenotypes of interest can be a limitation that prevents analysis at near genome wide saturation. Therefore, developing methods to positively select for phenotypes of interest is a powerful way to increase the depth of genomic coverage.

In addition to REMI and NTG mutagenesis, other powerful molecular techniques have been established for *Dictyostelium*. These include the 5-fluoroorotic acid (5-FOA)/*pyr56* counterselection, a method that has supported genetic investigations in *Dictyostelium* (Kalpaxis et al., 1991; Kuspa and Loomis, 1992; Insall et al., 1994; Gaudet et al., 2007). In this system, the prodrug 5-FOA is processed to the toxic form 5-fluorouracil by a uridine monophosphate (UMP) synthase enzyme (Kalpaxis et al., 1991). This allows for selection against cells with an active UMP synthase (Pyr56 in *Dictyostelium)* in the presence of 5-FOA. Alternatively, one can use the same system to select for cells with active Pyr56. In this case, cells are grown in the absence of uracil, a downstream product of Pyr56. In the absence of uracil, cells that do not have a functional copy of *pyr56* are selected against (Kalpaxis et al., 1991). 5-FOA screening can also be modified for monitoring specific phenomena. For example, in yeast attachment of aggregation prone regions such as amyloid-β (Aβ) can tune this system to measure the effect of protein phase separation on cellular fitness (Sanchez de Groot et al., 2019). Overall, this powerful counterselection system is highly useful in *Dictyostelium* research.

Having access to highly effective screening methodologies is vital to efficient genetic investigation. Previous work from our group and others identified that *Dictyostelium* was a proteostatic outlier that is highly resistant to polyglutamine aggregation (Malinovska et al., 2015; Santarriaga et al., 2015). To determine mechanisms that *Dictyostelium* utilize to resist polyglutamine aggregation, we performed a REMI screen to identify suppressors of polyglutamine aggregation in *Dictyostelium* (Santarriaga et al., 2018). While successful in identifying one suppressor of polyglutamine aggregation, the screen was limited in scope and did not approach genome wide coverage. This was largely due to the lack of a simple readout that required little labor coupled with the lack of any positive selection for hits of interest. Development of more efficient screening pipelines is therefore warranted to enable near genome wide saturation in reasonable time frames. Here we describe a novel screening pipeline that couples NTG mutagenesis with 5-FOA counterselection to robustly enrich for genes that suppress polyglutamine aggregation. We further discuss how this system can be modified to answer other biological questions.

## Materials and Equipment

Streptomycin (Gold Biotechnology, Cat. #S-150).

Carbenicillin (Gold Biotechnology, Cat. #SC-103).

Uracil (Alfa Aesar, Cat. #ch-0238).

CellTiter-Glo Luminescent Cell Viability Assay (Promega, Cat. #G7572).

DMSO (Gold Biotechnology, Cat. #SD-361).

5-FOA (Gold Biotechnology, Cat. #F-440).

NTG (Pfaltz & Bauer, Cat. #M23230).

*Dictyostelium discoideum* AX4 (Dictybase).

*Dictyostelium discoideum pyr56-*KO (developed here, available through Dictybase).

*Klebsiella aerogenes* (Dictybase).

pTX-GFP (Dictybase).

^Pyr56^mHtt^Q103,GFP^ plasmid (developed here, available through Dictybase).

α-GFP antibody (ThermoFisher Scientific, cat. # A-11122).

α-β-actin antibody [Iowa Developmental Studies Hybridoma Bank (DSHB)].

Enhanced chemiluminescence (ECL) buffer (GE Healthcare 28980926).

Tecan Spark M20 plate reader.

EVOS fluorescence microscope.

ChemiDoc imaging system (BioRad).

### Methods

#### General Culture

*Dictyostelium* cells were cultured at 22°C in HL5 medium supplemented with 300 μg/mL streptomycin (GoldBio cat. #S-150-100) and 100 μg/mL carbenicillin (GoldBio cat. #C-103-50). *pyr56-*KO cells were additionally supplemented with 20 μg/mL uracil (Alfa Aesar cat. #A15570). Where applicable, 5-fluoroorotic acid (GoldBio cat. #F-230-25) was added to the specified concentration. Cells were split regularly to maintain a density between 1 − 4 × 10^6^.

HL5 medium: 17.8 g proteose peptone (Millipore cat. #107229), 7.2 g yeast extract (BD cat. # 212720), 0.54 g Na_2_HPO_4_ (J. T. Baker cat. #3824-01), 0.4 g KH_2_PO_4_ (Sigma cat. #P9791-1KG), 130 μL B12/folic acid mix. Bring to 1 L with deionized water and adjust pH to 6.5 ± 0.1. Autoclave and add 20 mL of filter sterilized 50% glucose prior to use (Eichinger and Rivero-Crespo, 2006). B12/folic acid mix: 5 mg B_12_ (Sigma cat. #V2876-1G), 200 mg folic acid (Sigma cat. #F7876-10G). Add 95 mL deionized water and adjust pH to 6.5. Adjust volume to 100 mL, filter sterilize, and store at −20°C (Eichinger and Rivero-Crespo, 2006). FM minimal medium: 19.3 g premixed FM minimal medium (Formedium cat. #FMM0102). Add 1 L deionized water and filter sterilize.

#### Knockout Generation by CRISPR-Cas9

CRISPR-Cas9 knockout generation was completed as previously described for *Dictyostelium* (Sekine et al., 2018). Axenic *Dictyostelium* strain AX4 served as the wild-type starting strain for this gene knockout. Guide RNAs (gRNAs) against the *Dictyostelium pyr56* gene were developed using the CRISPR RGEN Tools Cas-Designer (Park et al., 2015) (Fwd: 5′ AGCATATCAAAAGGTTTATAAATC 3′; Rev: 5′ AAACGATTTATAAACCTTTTGATA 3′). These gRNAs were checked for off-target sites using the CRISPR RGEN Tools Cas-OFFinder (Bae et al., 2014).

#### Plasmid Construction

To develop the PTX(act15/pyr56-mHtt-GFP) plasmid, Htt exon 1 with 103 glutamines (sourced from Addgene plasmid #1385, the pYES2/103Q plasmid deposited by Michael Sherman) was first cloned into pTxGFP using *Kpn*I and *Xba*I (Levi et al., 2000; Santarriaga et al., 2015). The *pyr56* gene was PCR-amplified from AX4 genomic DNA and cloned into this construct using *Kpn*I. The *Kpn*I site within the *pyr56* gene was mutated using the QuikChange Lightning Site-Directed Mutagenesis Kit (Agilent Cat.# 210518), resulting in a synonymous t306c single nucleotide mutation that is silent at the amino acid level and removal of the internal restriction site. The Pyr56^GFP^ plasmid was constructed by cloning the t306c *pyr56* gene into pTxGFP using *Kpn*I.

#### Analysis of Viability

Cell viability was measured using the CellTiter-Glo Luminescent Cell Viability Assay from Promega (Cat. #G7572). To assess the sensitivity of wild-type *Dictyostelium* to 5-FOA, AX4 cells were washed and then resuspended in FM minimal media containing 0 μg/mL (DMSO) to 500 μg/mL 5-FOA. These cells were incubated in a stationary incubator at 22°C for 48 h. Cells were then mixed with the Cell-Titer Glo reagent according to manufacturer’s protocol, and luminescence was measured using a Tecan Spark M20 plate reader. Similarly, to confirm resistance to 5-FOA in the *pyr56-*KO strain, knockout cells in FM minimal media containing 20 μg/mL uracil were exposed to 0–500 μg/mL 5-FOA and incubated for 48 h at 22°C, followed by measurement with the CellTiter-Glo assay. To assess rescue of 5-FOA sensitivity, *pyr56-*KO cells were electroporated with the PTX(act15/pyr56-mHtt-GFP) plasmid. Following isolation of a clonal line from a bacterial lawn, *pyr56-*KO cells expressing ^Pyr56^mHtt^Q103,GFP^, AX4 wild-type, and *pyr56-*KO cells were exposed to either DMSO or 500 μg/mL 5-FOA. After 48 h, viability was measured using the CellTiter-Glo assay. Each of these viability experiments were performed in triplicate. Statistical analysis was performed using GraphPad Prism.

#### NTG Mutagenesis, 5-FOA Selection, Isolation of Clonal Populations

Chemical mutagenesis by NTG was performed as previously described (Li et al., 2016). Approximately 10^8^ *pyr56-*KO cells expressing ^Pyr56^mHtt^Q103,GFP^ were treated with 500 μg/mL NTG for 12 min in a shaking 22°C incubator. Following removal of NTG, cells were left to recover for 24 h. Cells were then treated with either DMSO or 1 mg/mL 5-FOA. Media was refreshed on days two and four following 5-FOA addition. Images taken after 5 days of culturing using an EVOS fluorescence microscope and quantification was performed using the ImageJ Cell Counter plugin to determine the number of cells with blinded, manual counting of the number of puncta.

5-FOA selected cells were collected for clonal isolation 5-days post 5-FOA addition. Clonal populations of mutagenized *Dictyostelium* were isolated from *Klebsiella aerogenes* bacterial lawns on SM agar plates. Isolated cells were then grown for 48 h before protein aggregation phenotype was recorded. Protein aggregation was observed as punctate GFP using an EVOS fluorescence microscope. Isolates with approximately five percent or more cells containing puncta were considered mutant for the protein aggregation phenotype.

#### SDS-PAGE and Western Blot Analysis

Lysates from *pyr56-*KO cells expressing empty vector (pTxGFP), Pyr56-GFP, and Pyr56-mHtt^Q103^-GFP were collected in boiling 1X Laemmli buffer followed by sonication for completion of cell lysis. Lysates were then run on a 4–20% gradient SDS-PAGE gel and transferred to polyvinylidene difluoride (PVDF) membrane for analysis by western blot. Membranes were blocked for 30 min with 5% milk in TBST, followed by overnight incubation with primary antibody at 4°C. Green fluorescent protein (GFP) expression was probed using polyclonal rabbit anti-GFP antibody at a 1:1,000 dilution in milk (ThermoFisher Scientific, cat. # A-11122) and beta actin expression was probed using a *Dictyostelium* specific monoclonal mouse anti-beta actin antibody at a 1:100 dilution (DSHB Hybridoma Product 224-236-1, deposited to the DSHB by Gerisch, G.). Membranes were washed with TBST three times for 10 min at room temperature before and after incubation with secondary antibody for 1 h. For development, membranes were incubated in enhanced chemiluminescence (ECL) buffer for 2 min at room temperature then imaged using a ChemiDoc imaging system.

## Results

**FIGURE 1:**
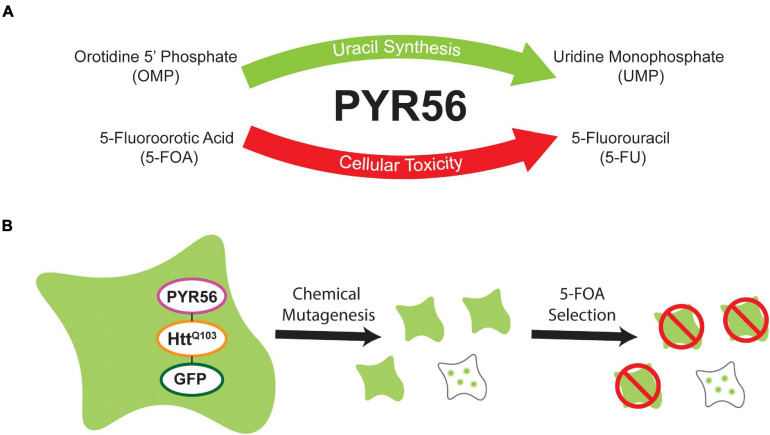
Overview of selection strategy. **(A)** The *Dictyostelium* UMP synthase, Pyr56, is an essential component of the *de novo* uracil synthesis pathway. However, Pyr56 also catalyzes the conversion of the prodrug 5-FOA to toxic 5-FU, leading to cell death. **(B)** A fusion protein containing the UMP synthase Pyr56 fused to a polyglutamine-expanded fragment of Htt exon 1 and a fluorescent reporter GFP will remain soluble in *pyr56-*KO cells. Following chemical mutagenesis, cells which lose function of a polyglutamine aggregation suppressing factor will demonstrate aggregation of the fusion protein. Sequestration of Pyr56 activity by protein aggregation confers resistance to 5-FOA, allowing for selection of polyQ aggregation mutants.

*Dictyostelium* is a tractable model system that can be utilized to gain insight into processes that regulate human health and disease. One advantage of *Dictyostelium* is that genetic tools exist to allow near genome saturation screens, allowing one to identify genes that participate in pathways of interest. While creating libraries of mutated *Dictyostelium* is rapid and efficient, downstream processes like clonal cell isolation can be time consuming, leading to constraints on the depth of genome coverage obtained. This is largely because a low percentage of cells will be positive for a given phenotype and the fact that clonal isolation is tedious. This is exacerbated in screens that rely on readouts like fluorescence which require isolation of clones, removal of bacteria, clonal outgrowth, and imaging. To overcome these issues, we sought to develop a selection system that would enhance for phenotypes of interest, in our case the presence of aggregated GFP puncta, thus eliminating the majority of cells lacking the desired phenotype and subsequently increasing throughput (Figure 1).

**FIGURE 2:**
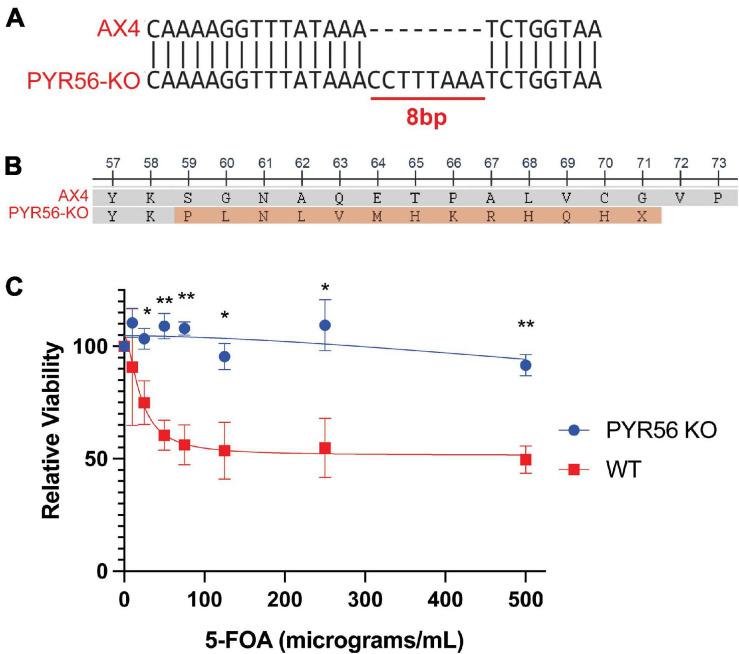
Deletion of *pyr56* confers resistance to 5-FOA toxicity. **(A)** The *pyr56-*KO generated by CRISPR-Cas9 contains an 8 bp insertion in the Pyr56 gene. **(B)** The 8 bp insertion in the Pyr56 gene results in a frameshift after amino acid residue 58 and a premature stop after residue 71. **(C)** Deletion of *pyr56* results in survival when cells are grown in media containing 5-FOA. Either wild-type or *pyr56-*KO *Dictyostelium* at a starting concentration of 5 × 10^3^ cells/mL were grown in presence of increasing concentrations of 5-FOA. Viability was measured by luminescence using the CellTiter-Glo Viability Assay at 48 h (*n* = 3). Samples were analyzed for statistical significance using an unpaired *t*-test, *p < 0.05. **p < 0.001.

To accomplish this, we utilized the 5-FOA counterselection system (Kalpaxis et al., 1991). This system is ideal in *Dictyostelium* as the genome encodes a single copy of the uridine monophosphate (UMP) synthase enzyme, Pyr56. To enable 5-FOA screening, we generated a *pyr56* knockout strain (*pyr56*-KO) using CRISPR-Cas9. In this strain, there is an 8 bp insertion after amino acid residue 58, resulting in a frame shift and introduction of a premature stop codon after residue 71 (Figures 2A,B). Importantly, the *pyr56-*KO strain was viable and resistant to 5-FOA toxicity (Figure 2C). It is worth mentioning that cells may escape selection when grown in monolayer culture, as noted in the original characterization of the *pyr56*/5-FOA selection system (Kalpaxis et al., 1991). Thus, one may want to consider culturing conditions when determining the required strength of selection.

**FIGURE 3:**
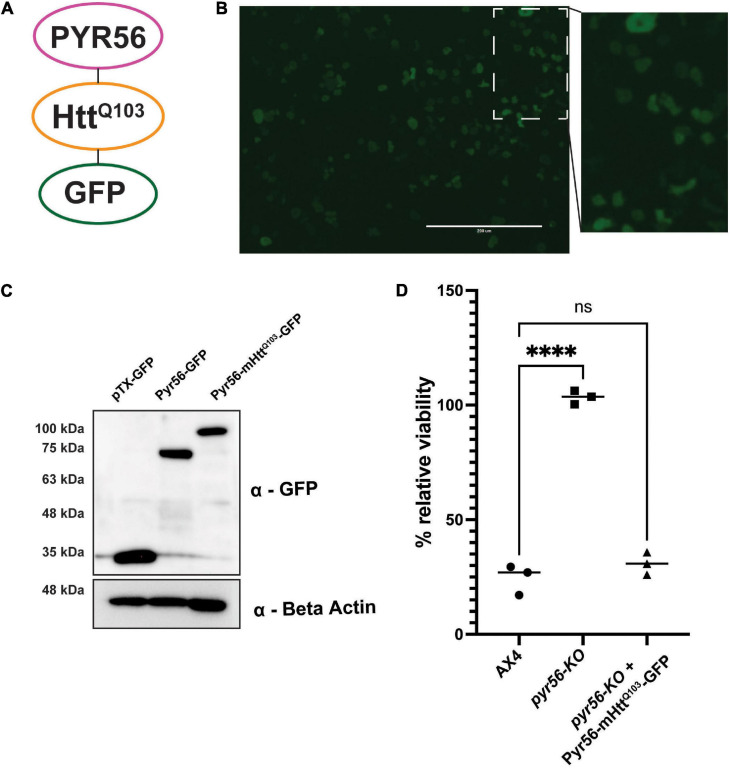
The Pyr56-mHtt^Q103^-GFP fusion protein is soluble and active in *Dictyostelium*. **(A)** The Pyr56-mHtt^Q103^-GFP fusion protein is composed of the UMP synthase Pyr56, Htt exon 1 containing a tract of 103 glutamines, and a GFP reporter express by the PTX(act15/pyr56-mHtt-GFP) plasmid. **(B)** Pyr56-mHtt^Q103^-GFP remains soluble in *Dictyostelium*. *pyr56-*KO cells expressing Pyr56-mHtt^Q103^-GFP were imaged for GFP localization using an EVOS microscope. Scale bar represents 200 μm. **(C)** Pyr56-mHtt^Q103^-GFP is expressed in *Dictyostelium*. *pyr56-*KO cells expressing either empty vector (pTxGFP), Pyr56^GFP^ or Pyr56-mHtt^Q103^-GFP were lysed, and lysates were run on a gradient SDS-PAGE gel. Gels were analyzed by western blot analysis to probe for GFP expression, indicating that Pyr56-mHtt^Q103^-GFP is expressed in *Dictyostelium* and runs at the expected size. **(D)** Expression of Pyr56-mHtt^Q103^-GFP in the *pyr56-*KO strain rescues sensitivity to 5-FOA. 2 × 10^3^ cells/mL of either wild-type, *pyr56-*KO, or *pyr56-*KO expressing Pyr56-mHtt^Q103^-GFP were treated with 500 μg/mL 5-FOA and viability was measured at *t* = 48 h by luminescence using the CellTiter-Glo Viability Assay (*n* = 3). Samples were analyzed by one-way ANOVA with multiple comparisons, ^∗∗∗∗^ indicates *p* < 0.0001.

In our initial screen to identify suppressors of polyglutamine aggregation, we isolated clonal populations of *Dictyostelium* and screened *via* high content imaging. This required screening large numbers of colonies as the number of hits was low. To greatly enhance the number of hits, we next wanted to develop a system that positively select for *Dictyostelium* cells that contained polyglutamine aggregates. To accomplish this, we cloned the Pyr56 protein as a fusion with a fragment of mutant huntingtin protein containing a homopolymeric repeat of 103 glutamines and GFP [pTX(act15/pyr56-mHtt-GFP)] to express the Pyr56-mHtt^Q103^-GFP protein (Figure 3A). This construct was introduced into *Dictyostelium* cells using pTX-GFP, an extrachromosomal multi-copy plasmid. This is important because resistance to 5-FOA could arise from mutations in the exogenous *pyr56* gene rather than sequestration of Pyr56 activity by protein aggregation. However, even if this were to occur, it is unlikely that it would result in false positives as it would likely accumulate as soluble protein rather than aggregated protein and therefore not be identified as a hit in our screen. Using this construct, we confirmed that Pyr56-mHtt^Q103^-GFP is expressed and soluble in *Dictyostelium* and migrates at the expected molecular weight when analyzed by western blot (Figures 3B,C). Importantly, Pyr56-mHtt^Q103^-GFP expressed by the PTX(act15/pyr56-mHtt-GFP) plasmid is active and is capable of restoring toxicity in the presence of 5-FOA (Figure 3D).

**FIGURE 4:**
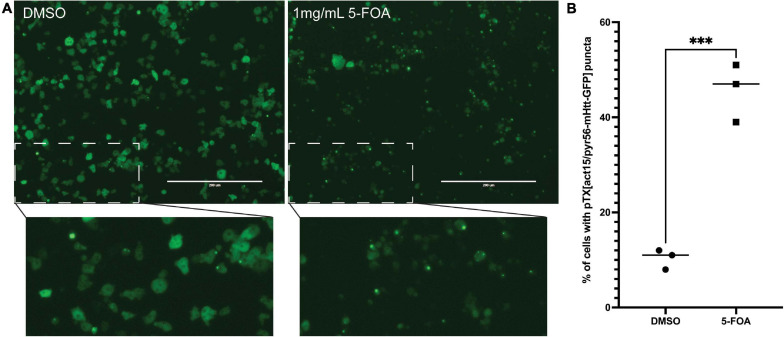
Selection of NTG mutagenized cells with 5-FOA enriches for protein aggregation mutants. **(A)** Mutagenized cells treated with 5-FOA are enriched for punctate GFP. *pyr56-*KO cells expressing ^Pyr56^mHtt^Q103,GFP^were treated with NTG and allowed to recover for 24 h following mutagenesis. After recovery, cells were treated with either DMSO control (left) or 1 mg/ml 5-FOA (right). Five days after 5-FOA addition, there is clear enrichment for cells containing GFP puncta in the 5-FOA treated cells when compared to the DMSO control which contains a majority of cells with diffuse GFP. This indicates enrichment of polyQ aggregation mutants in the population of 5-FOA selected cells. Scale bar represents 200 μm. **(B)** Average number of cells containing puncta. Cell number and percentage of cells containing Pyr56-mHtt^Q103^-GFP were quantified using the ImageJ Cell Counter (*n* = 3). Samples were analyzed by an unpaired *t*-test, ^∗∗∗^ indicates *p* = 0.0007.

**FIGURE 5:**
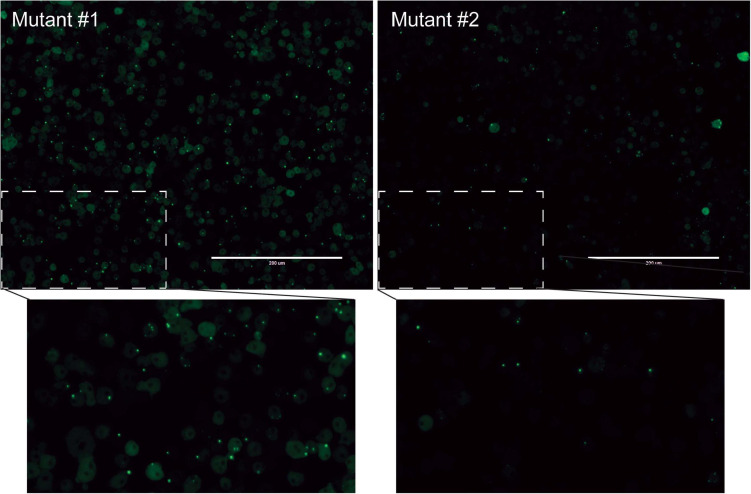
Two protein aggregation mutants obtained using this selection technique. Mutagenized and 5-FOA selected cells were plated on SM agar plates containing bacterial lawns. Colonies were isolated following plaque formation. After 48 hours of growth, cells were analyzed for GFP puncta using an EVOS microscope.

We next wanted to utilize the PTX(act15/pyr56-mHtt-GFP) construct to develop a screen that positively selects for *Dictyostelium* cells containing mutations in genes that suppress polyglutamine aggregation. To accomplish this, we performed NTG mutagenesis as previously described on *pyr56-*KO *Dictyostelium* expressing Pyr56-mHtt^Q103^-GFP (Li et al., 2016). These cells were grown for 24 h prior to addition of either DMSO or 1 mg/mL 5-FOA for 5 days. At the end of 5 days, we observed a clear enrichment of *Dictyostelium* that contain Pyr56-mHtt^Q103^-GFP aggregates (Figure 4A). To determine the percentage of *Dictyostelium* cells positive for the protein aggregation phenotype, we analyzed 3 populations of mutagenized cells that had been treated with either DMSO or 1 mg/mL 5-FOA using fluorescence microscopy and found a significant increase in the number of cells with Pyr56-mHtt^Q103^-GFP aggregates (Figure 4B). To isolate clonal populations of mutant *Dictyostelium*, we plated 5-FOA selected *Dictyostelium* cells on bacterial lawns and picked individual colonies following plaque formation. Representative images of mutant isolates of cells with polyglutamine aggregates are shown (Figure 5). With this system, screening just 500 colonies is expected to yield hundreds of desired mutants. Following isolation of desired mutants, these strains can be subjected to whole genome sequencing and variant analysis to identify potential causative mutations for further investigation (Li et al., 2016).

## Discussion

### Summary of Findings

Here we have developed a highly efficient genetic screening workflow for *Dictyostelium* that allows for positive or negative selection of hits (Figure 1). We utilized CRISPR-Cas9 to develop a *Dictyostelium* strain that lacks *pyr56*, the gene that encodes for *Dictyostelium’s* UMP synthase gene and demonstrated that the *pyr56-*KO strain is resistant to 5-FOA induced toxicity (Figure 2). We also constructed a vector for overexpression of Pyr56-mHtt^Q103^-GFP and demonstrated that this protein was soluble and induced toxicity in the presence of 5-FOA (Figure 3). We further demonstrated that sequestration of Pyr56 activity by protein aggregation coupled with selection by 5-FOA resulted in an increase in *Dictyostelium* cells that have Pyr56-mHtt^Q103^-GFP puncta (Figures 4,5). Together these experiments outline high throughput screening strategies that offer negative selection to greatly enrich hit rate and reduce the number of clones that must be screened.

### Utilization of This Platform for Assessing Other Biological Systems

In addition to being useful for screening for proteins that suppress polyglutamine aggregation this screening modality can be modified for use in other systems. For example, *Dictyostelium* has been reported to have a proteome extremely rich in prion-like proteins (Eichinger et al., 2005); however, virtually nothing is known about factors that regulate prion-like proteins in *Dictyostelium*. Using this screening workflow, one could both identify factors that promote and suppress aggregation of prion-like proteins. Similarly, one could use this system to investigate regulators which interact with structurally distinct protein domains that present similar aggregation phenotypes. These regulators include various categories of RNA which are becoming increasingly studied in the context of phase separation (Guo et al., 2021). For example, this screening methodology could also be used for investigating the role of long non-coding RNAs (lncRNAs) in phase separation. lncRNAs can sequester proteins into foci to inhibit their function and little is known about factors that regulate lncRNA function (Luo et al., 2021). Genetic screens to identify factors that modulate lncRNA function could provide new insight into lncRNA biology.

While many proteins are known or believed to phase separate, the exact behavior of these proteins within the cell can be difficult to observe. However, certain characteristics of the selection system we have presented here may be adapted to improve the techniques available to investigate this phenomenon. For instance, given that aggregation of URA3-GFP-Aβ is linked to cellular fitness for yeast in 5-FOA enriched media (Sanchez de Groot et al., 2019), one can extrapolate that sequestration of essential Pyr56 activity into a phase-separated compartment is directly related to cellular growth in *Dictyostelium*. Thus, given that protein aggregation is correlated with cellular growth in these conditions, the techniques described here could be adapted to quantitatively estimate relative amounts of phase separated protein *in vivo* using *Dictyostelium*.

Selection by 5-FOA has recently been applied to human cell lines (Wiebking et al., 2020). While this selection system has not yet been widely investigated in human cells, one can envision how our screening methodology could be applied to investigate factors which influence protein aggregation and phase separation in human cells. Thus, beyond translational relevance from model organisms, our technique has great potential for understanding human health in aspects such as neurodegeneration, aging, and many more.

### Uracil Auxotrophy Can Be Utilized for Selection

In addition to negatively selecting against Pyr56 activity, this system could also be altered to positively select for soluble, active Pyr56. In this case, one would exploit the fact that the *pyr56-*KO strain is a uracil auxotroph that requires the presence of uracil to grow. Here, a lack of uracil in the media would positively select for clones that have soluble, active Pyr56 protein. Coupled with NTG mutagenesis or REMI screening, this could be useful for identifying genes that negatively regulate cellular processes where proteins reversibly form puncta. Such a screen could be useful for proteins that prevent processes like liquid-liquid phase separation (LLPS) or promote the resolution of biomolecular condensates. This would be a useful screen as LLPS has recently been identified in *Dictyostelium* (Santarriaga et al., 2019). More recent work has also demonstrated that one type of biomolecular condensate, stress granules, are dynamically modulated and that different stressors alter which factors are needed to resolve the stress granules once the stressor has been removed (Gwon et al., 2021; Maxwell et al., 2021). Furthermore, LLPS has also been investigated recently in yeast as a tunable mechanism of responding to environmental changes, thus conferring a fitness benefit under certain stressful conditions (Riback et al., 2017; Franzmann et al., 2018; Franzmann and Alberti, 2019; Lau et al., 2020). This suggests that genetic screens in *Dictyostelium* may serve as one mechanism to identify genes that regulate stress granule dynamics and cellular responses to environmental stressors.

## Data Availability Statement

The raw data supporting the conclusions of this article will be made available by the authors, without undue reservation.

## Author Contributions

FW and KS developed and planned the project, interpreted the results, and wrote the manuscript. FW and YW performed the experiments. KS supervised the research. All authors reviewed and edited the manuscript.

## Conflict of Interest

The authors declare that the research was conducted in the absence of any commercial or financial relationships that could be construed as a potential conflict of interest.

## Publisher’s Note

All claims expressed in this article are solely those of the authors and do not necessarily represent those of their affiliated organizations, or those of the publisher, the editors and the reviewers. Any product that may be evaluated in this article, or claim that may be made by its manufacturer, is not guaranteed or endorsed by the publisher.
